# Limitations of Weight Velocity Analysis by Commercial Computer Program *Growth Analyser Viewer Edition*

**DOI:** 10.1007/s10439-018-02118-8

**Published:** 2018-08-27

**Authors:** Martin J. C. van Gemert, Cornelis M. A. Bruijninckx, Ton G. van Leeuwen, H. A. Martino Neumann, Pieter J. J. Sauer

**Affiliations:** 1 Department of Biomedical Engineering & Physics, Amsterdam UMC, University of Amsterdam, Meibergdreef 9, 1105 AZ Amsterdam, The Netherlands; 2 Surgery Expert Witness, Rotterdam, The Netherlands; 3 Department of Dermatology, Erasmus University Medical Center, Rotterdam, The Netherlands; 4 Department of Pediatrics, University Medical Center, Groningen, The Netherlands

**Keywords:** Weight gain of infants, Standard weight curve, Analysis of weight growth from weight curves, Model simulations, Weight velocity patterns

## Abstract

Commercial software package “Growth Analyser Viewer Edition” (“GAVE”) aims to document, monitor and analyze growth and development in children and adolescents. Although its clinical and scientific use is widespread, there are no published studies that describe the method and its validation. We were informed that GAVE calculates the weight velocity (kg/year) at age *t* from the weight difference between *t* and 448 days earlier or at birth, divided by the time difference. We recently discussed a case of false child abuse diagnosis (Pediatric Condition Falsification), resulting in the separation of the child from its parents, in which GAVE played a negative contributing role. To prevent such inappropriate diagnoses, we analyzed GAVE from a schematic representation of the measured clinical weight curve, with precisely defined weight velocities. In conclusion, the 448 days included for weight velocity predictions by GAVE caused the erroneous outcomes. Until the necessary changes to the software are implemented and validated, we advise against the use of GAVE in infants younger than 1.5 years, if multiple weight changes occur within 448 days, and following a long-lasting weight velocity change. Our analysis suggests to discard all medical software packages that lack public description and proof of validation.

## Introduction

Growth of infants, such as gain in weight and height, is a key element in their development. Growth according to normal standards is considered a sign of health while abnormal growth may indicate an illness. Methods to identify abnormal growth are therefore much needed. The *Dutch Growth Research Foundation*^10^ offers medical professionals, researchers, patients and parents a collection of software products called *Growth Analyser* to *easily document*, *monitor and analyse the growth and development of children and adolescents*. One variant of Growth Analyser, *Growth Analyser Viewer Edition*, abbreviated in this paper as “GAVE”, is an add-on for electronic hospital record systems.^10^

Although GAVE has been used both in clinical settings and scientific research, we could neither identify a publication of the method nor of its validation. We obtained information about the algorithm of this program from personal communication with one of the developers. In this paper we limit ourselves to the calculation of the weight gain achieved during a specific age period divided by that period, called *weight velocity* (expressed in kg/year). We also limit ourselves to measured weights in infants aged less than three years as this is part of a period in which weight gain can vary from strongly positive to even negative values.

We recently published a case report,^15^ briefly summarized in the Appendix, of a boy born after an uneventful pregnancy with a birthweight of 3.18 kg, which coincides with the − 0.6SD or P25 standard weight curve. Abbreviations SD and *P* stand for standard deviation and percentile; P25 expresses that 25% of the children have a weight below- and 75% above this weight curve. The boy’s mother was falsely accused of a rare form of child abuse, called Munchausen Syndrome by Proxy or Pediatric Condition Falsification,^5^ which led to the separation of the child from his family for 8 months. The boy was diagnosed with Failure-to-Thrive, a diagnosis that requires an insufficient weight *gain*,^12^,^13^ assessed by comparing actual weight gain with weight gain based on standard weight curves, e.g., as tabulated by Gerver and de Bruin.^8^ The clinical sign Failure-to-Thrive is usually seen in infants of less than 4 years of age. The use of GAVE played an important but negative role during a legal appeal procedure of the parents against the separation verdict because it contributed to continuation of the foster home period. Although the initial suspicion of Pediatric Condition Falsification was not based on an analysis of weight velocity by GAVE, it was confirmed, incorrectly, by using GAVE, see sixth paragraph of Discussion. On closer inspection, we found that GAVE calculated erroneous weight velocities, with significant under- as well as overestimations, during virtually the whole time frame.

In this paper, our focus is on analyzing the method of weight velocity calculations by GAVE by means of three weight curves: the 0SD curve, the clinical weight curve and a schematic model representation of this clinical curve. Our aims are, first, to identify the source of the invalid weight gain analysis by GAVE and, second, to determine the mechanisms that are responsible for producing these erroneous weight velocities.

Our presentation is as follows. First, we (i) demonstrate the method using the 0SD curve, (ii) present the clinical weight curve,^15^ and (iii) its schematic model representation based on period-averaged-weight-velocities of the clinical weights. Second, we compare the weight velocities calculated by GAVE with the exact weight velocities of (1) the 0SD curve and (2) the schematic weight model.

## Materials and Methods

Weight velocity at age *t*, calculated by GAVE, and expressed in kg/year, is defined as the difference between the weight at age *t* and the weight at 448 days earlier, called the “earlier weight”, divided by 448/365.25 = 1.23 years, assuming one year is 365.25 days. When a weight measurement is not available on day (*t * – 448), the next available weight is used at (*t* – 448 + *ɛ*). When age *t* is less than 448 days, GAVE uses the weight gained since birth divided by age *t*/365.25. Thus

1a $${\text{Weight}}\;{\text{velocity}}\; (t < 448) = \frac{{{\text{Weight}}\; (t )- {\text{Birthweight}}\;}}{t/365.25}\quad t < 448\,{\text{days}}, $$

1b $$ {\text{Weight}}\;{\text{velocity}}\; (t \ge 448) = \frac{{{\text{Weight}}\; (t) - {\text{Weight}}\; (t - 448 + \varepsilon )}}{1.23}\quad t \ge 448\,{\text{days}} . $$

**Figure 1:**
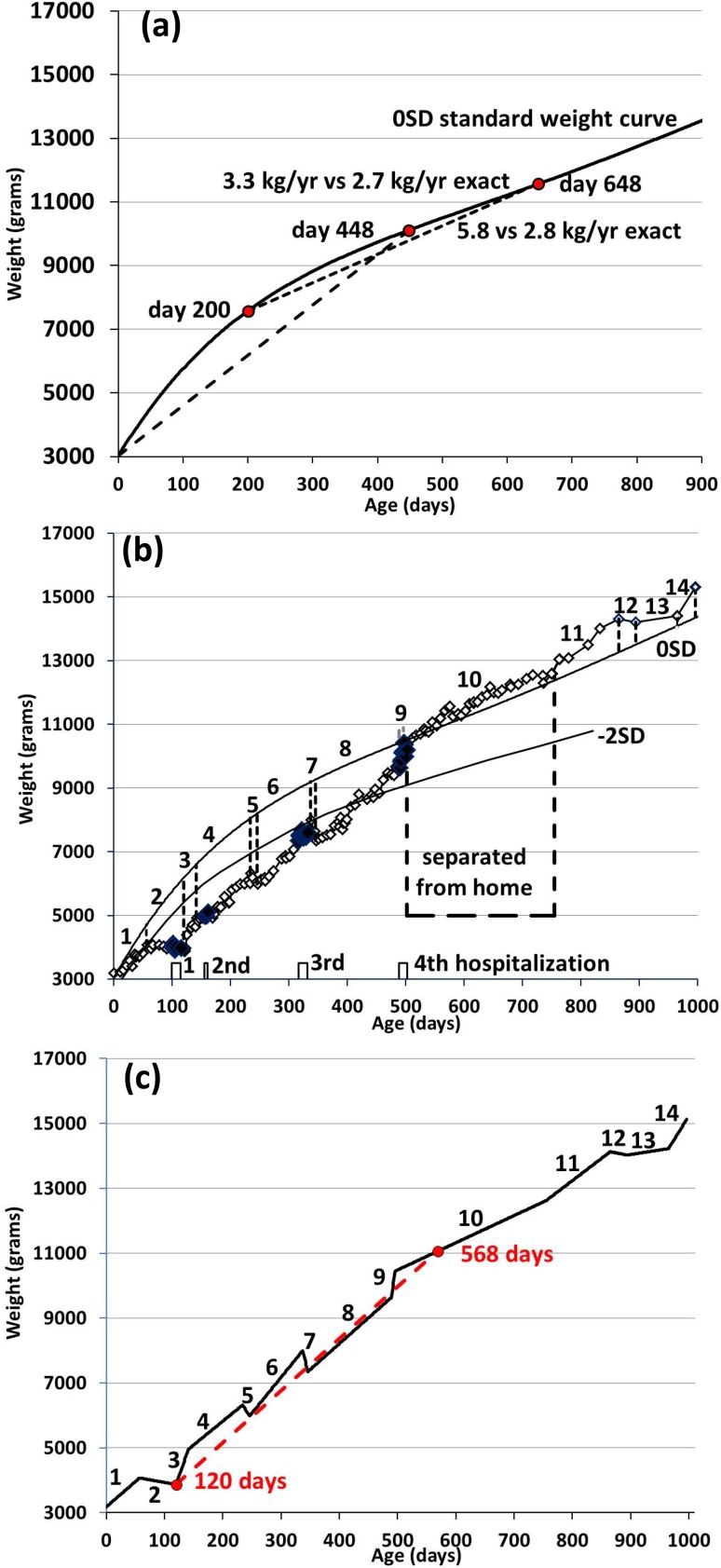
Three weight curves. (**a**) Standard weight curve 0SD (P50) for Dutch boys,^8^ and two examples of weight velocity calculation by GAVE, at day 448, where the birthweight is the earlier weight, and at day 648, where the weight at day 200 is the earlier weight. We fitted the weight data from Ref.^8^ by a fifth degree power series of age in Excel. (**b**) Measured weights (open lozenges, hospitalizations by thick black lozenges),^15^ the 0SD and − 2SD weight curves until 996 days of age divided into 14 consecutive periods. (**c**) Schematic model of (**b**) using the 14 period-averaged-weight-velocities of (**b**), and an extreme example of weight velocity assessment at age 568 days, with earlier weight at 120 days.

Figure 1a demonstrates GAVE’s calculation of weight velocity at 448 days, where the birthweight is the earlier weight, and at 648 days, where the weight at 200 days is the earlier weight. We used the 0SD or P50 standard weight curve for Dutch boys.^8^

We determined the period-averaged-weight-velocities of a clinical case of weight measurements,^15^ divided into 14 consecutive age periods during 996 days (Fig. 1b). Previously, we used eight non-consecutive periods until 865 days of age.^15^ These 14 periods were chosen because of their reasonably linear weight vs. age behavior so we could characterize them by linear trendlines in Excel 2010. We also included the 0SD and − 2SD standard weight curves as before.^15^ We connected these 14 consecutive linear trendlines in the analysis as a schematic model description with precisely defined weight velocities over all 14 age periods (Fig. 1c and Table 1). We approximated the periods between the four hospitalizations and separation from home as single growth lines except between the second and third hospital period where we included two weight dips. This approach allows an accurate test of the predictions by GAVE. We acknowledge that our choice of 14 periods is somewhat arbitrary to model the clinical data of Fig. 1b but it is adequate for the purpose of comparing GAVE’s predictions with exact weight velocities.

**Table 1:** Weight gain and exact vs. GAVE-derived weight velocities in 14 age periods.

Period (days)	Figure 1b		Figure 1c		Figure 2a
	Weight gain (kg)	Period-averaged-weight-velocity (kg/year)	Weight gain (kg)	Schematic weight velocity (kg/year)	GAVE weight velocity range (kg/year)
1 (0–56)	0.89	5.7	0.874	5.7	5.7
2 (56–120)	− 0.2	− 1.14	− 0.184	− 1.05	5.7–2.1
3 (120–141)	1.056	17.1	1.09	19.0	2.1–4.6
4 (141–234)	1.36	5.5	1.36	5.3	4.6–4.9
5 (234–246)	− 0.34	− 9.5	− 0.34	− 10.3	4.9–4.2
6 (246–337)	2.015	7.5	2.015	8.1	4.2–5.2
7 (337–346)	− 0.645	− 25.5	− 0.645	− 26.2	5.2–4.4
8 (346–489)	2.28	6.2	2.28	5.8	4.4–4.7
9 (489–496)	0.82	45.0	0.82	42.8	4.7–5.3
10 (496–755)	2.172	3.55	2.172	3.1	5.9–4.3
11 (755–865)	1.547	5.12	1.506	5.0	4.1–4.7
12 (865–894)	− 0.104	− 1.31	− 0.104	− 1.31	4.6–4.2
13 (894–965)	0.2	1.02	0.2	1.02	4.2–2.9
14 (965–996)	0.9	10.6	0.9	10.6	3.0–3.5

**Figure 2:**
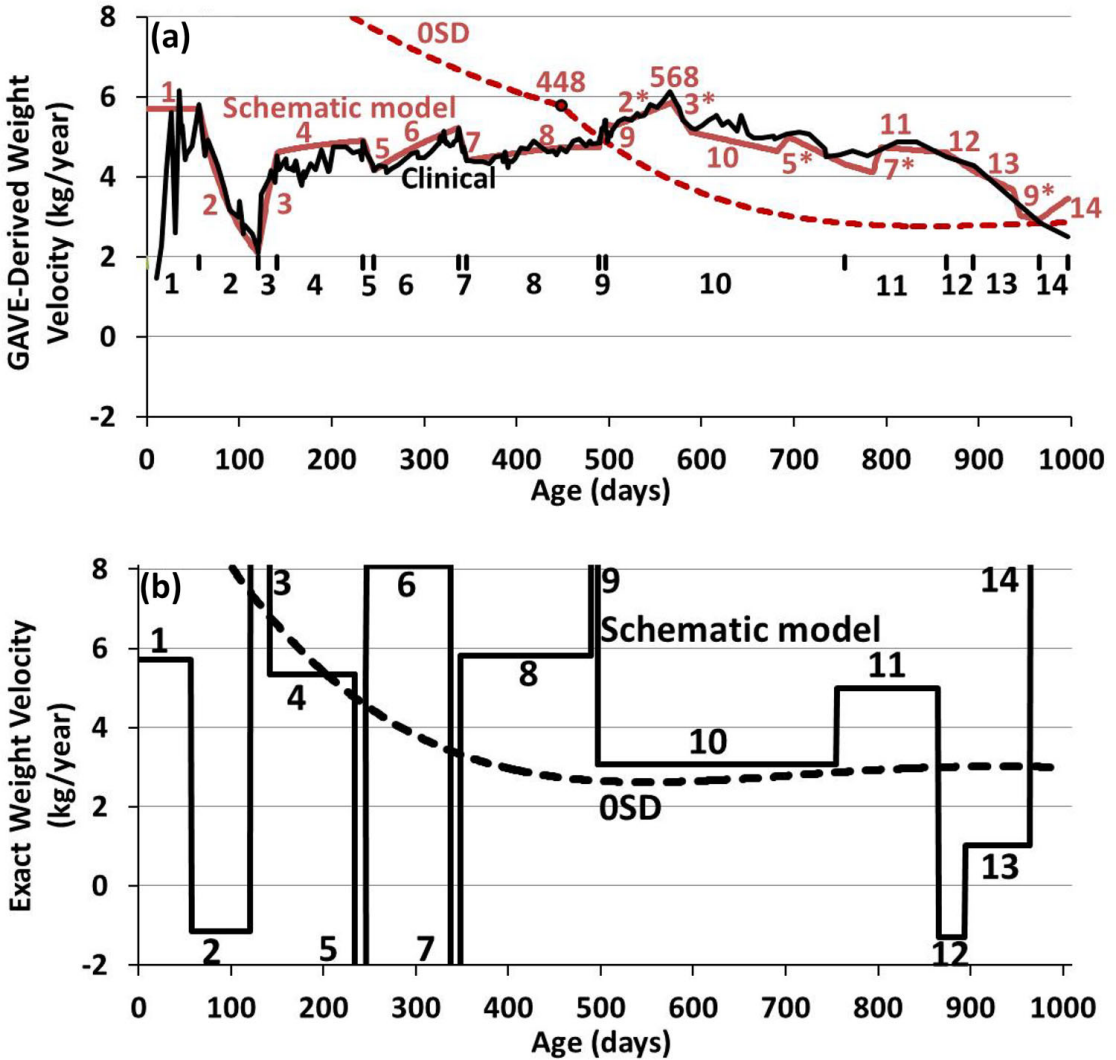
GAVE vs. exact weight velocities. (**a**) Response by GAVE of the 0SD (dashed brown line), clinical case (black line) and schematic model (brown line) in the 14 age periods indicated with brown numbers and on the horizontal line *y* = 2. Inverse-echoes of abrupt effects that occurred 448 days earlier are identified by an asterisk (“*”). The maximum of the inverse-echoes 2* and 3* is at day 568 (Fig. 1c). Note that GAVE totally misses a negative weight velocity (Failure-to-Thrive period 2) and that inverse echoes 2*, 3* and 5* also contribute to a seriously overestimated weight velocity during separation period 10. Inverse echo 7* causes an increased accuracy of GAVE’s period 11. (**b**) Exact schematic weight velocities from Fig. 1c (black line) (details in Table 1), and of the 0SD curve obtained from Eq. (2) (dashed black line).

During each of 14 periods (column 1), the weight gain, the clinical period-averaged-weight-velocity (column Fig. 1b, assessed by linear trendlines in Excel), and the schematically included weight velocity (column Fig. 1c), vs. GAVE’s range of weight velocity predictions (column Fig. 2a). Period 2 denotes Failure-to-Thrive. Period 10 denotes separation from home

The 0SD curve (Fig. 1a),^8^ was fitted in Excel to a fifth-order function of age. Its exact weight velocity was determined by numerical differentiation according to

2 $$ {\text{Exact}}\;{\text{weight}}\;{\text{velocity}}\; (t) = \frac{{{dWeight}}\;(t)}{dt} = \frac{{{\text{Weight}}\; (t )- {\text{Weight}}\; (t - 1)}}{1}, $$

where age *t* is given in days separated by 1 day.

## Results

### Response Patterns of GAVE

Figure 2a shows the GAVE-predictions of the 0SD, the clinical and the schematic model weight curves for each of the 14 periods. Figure 2b shows the exact weight velocities of the 0SD and the schematic model. See also Table 1.

### 0SD Weight Curve

For the 0SD curve, Figs. 2a and 2b show that GAVE seriously overestimates the weight velocities from about 100 to 600 days, the age period that a healthy infant changes its weight relatively rapidly. A 2-tailed Mann–Whitney *U* test confirmed that these two curves are significantly different, to the level of *p* < 0.00001. Actually, the level of overestimation between days 170 and 550 is by a factor varying between 1.5 and 2.1, implying that such high but incorrectly predicted 0SD weight velocities can result in erroneously classifying the weight gain of an infant as inadequate, see, e.g., the sixth paragraph of the Discussion. At 448 days, a significant change in the time derivative of the GAVE-derived weight velocity occurs. Reasonably correct weight velocities by GAVE are reached at about 2 years of age.

### Clinical and Schematic Model Weight Curves

For the clinical and schematic model weights, Figs. 2a and 2b show the GAVE calculations to be erroneous over virtually the whole age period, resulting in significantly underestimated weight velocities during periods 3, 4, 6, 8, 9 and 14 as well as significantly overestimated weight velocities during periods 2, 5, 7, 10, 12 and 13, also completely missing the periods of actual weight loss, e.g., during the Failure-to-Thrive period 2. Reasonably correct clinical weight velocity predictions happened only during the second parts of periods 1 and 11, albeit that this latter prediction was positively affected by the inverse-echo of line 7, denoted as 7* in Fig. 2a, see the paragraph below on the response of GAVE for *t* ≥ 448 days.

A 2-tailed Mann–Whitney *U* test showed significant differences, to the level of *p *= 0.0064 for period 1 and *p* = 0.00512 for period 11, between the exact weight velocities and GAVE’s predictions of Fig. 1b. Similarly, this test also showed significant differences between the exact values and GAVE’s predictions of periods 1, 4 and the second part of period 11 of Fig. 1c, to the level of *p* < 0.00001. Because these chosen periods have the closest proximity between GAVE predictions and exact weight velocities, a significant difference between the two GAVE analyses (Fig. 2a) and their exact values in Fig. 2b has been established.

The weight velocity behaviors derived by GAVE from Figs. 1b and 1c shown in Fig. 2a, are complex and consist of very different weight velocity patterns that particularly but not only depend on whether weight velocity determination at age *t* occurs before or after 448 days.

### Response of GAVE to a Weight Change at *t* < 448 Days

For weight velocity determination, all ages until 448 days have one and the same earlier weight, the birthweight. From Eq. (1a), the weight velocity at age *t* from GAVE implies that if a significant change in weight occurs during a short age period, which is usually negative, in period 9 also positive,^15^ see Appendix, the response of GAVE is a change in weight velocity with the same sign as the weight change and during the same period. This can be seen from brown lines 2, 3, 5 and 7 in Fig. 2a. The subsequent time response of the weight velocity, which follows the behavior of the weight in the succeeding period, can be relatively fast, e.g., curves 2 and 3, or relatively slow, e.g. curves 4, 6 and 8 of Fig. 2a. When the weight curve changes its weight velocity while remaining constant over a significant time, e.g., periods 10 and 11 in Fig. 1c, the theoretical response of the weight velocity is a gradual decay towards the last weight velocity that takes 448 days, unless other changes, e.g., an inverse-echo, explained below around Eq. (3), occur earlier.

Equation (1a) also shows that the weight velocity amplitude is inversely proportional to its time of onset, *t*. This is the reason that the change in weight velocity during period 9, occurring around 495 days, is smaller than during periods 5 (around 235 days) and 7 (around 340 days), despite that 9 has the larger weight change amplitude (Fig. 1c and Table 1). See also Eq. (4) below.

### Response of GAVE to a Weight Change at *t* ≥ 448 Days

If a weight change occurs at an age beyond 448 days, the weight velocity response of GAVE can become very complex, even though the method itself is computationally not complex, because it depends on the weight at age *t* but also on the weight at 448 days earlier, at age (*t* − 448). We will consider two different conditions. First, the change in weight is predominantly at the actual age *t *> 448 days and much less at the earlier age (*t* − 448). Then, the weight velocity pattern by GAVE is in concept similar to that of the previous paragraph except that the inverse age dependence is absent here. Thus, the variation in weight velocity has the same sign as the weight changes and occurs during the same age period. Second, the change in weight is predominantly at the earlier age (*t *−448) and much less at the actual age of weight velocity determination *t *> 448 days, a condition leading to an “inverse-echo” response. Assuming for convenience here that the weight very close to earlier age (*t *−448) changed abruptly by a negative amount of − Δ*W* at age (*t* – 448 + *δt*), where *δt* ≪ *t*, vs. for this example no weight change close to the age of weight change determination (*t *+ *δt*), then the weight velocity at (*t *+ *δt*) is

3 $$ \begin{aligned} {\text{Weight}}\;{\text{velocity}}\; (t + \delta t)  = \frac{{{\text{Weight}}\; (t + \delta t) - {\text{Weight}}\; (t + \delta t - 448)}}{1.23} \\  = \frac{{{\text{Weight}}\; (t) - [{\text{Weight}}\;(t - 448) - \Updelta W]}}{1.23} \\ = {\text{Weight}}\;{\text{velocity}}\; (t) + \frac{\Updelta W}{1.23}. \\ \end{aligned} $$

Thus, a *negative* abrupt change in weight at age (*t *+ *δt* − 448) causes a *positive* echo in weight velocity at age (*t *+ *δt*), which we called “inverse-echo”. We emphasize that (*t *+ *δt*) can also represent the next available weight measurement. This example demonstrates that a weight pattern consisting of significant weight variations, e.g., lines 1–3, and lines 5, 7 and 9 in Fig. 1c, will be inversely echoed by GAVE exactly 448 days later (Fig. 2a). Inverse echoes are indicated by an asterisk, i.e., lines 2*, 3*, 5*, 7* and 9* are the inverse-echo of lines 2, 3, 5, 7 and 9, respectively.

Figure 2a also shows that inverse-echo 7* from weight dip 7 is only 40 days after day 755, the day the boy returned home, affecting the natural decay of the weight velocity response of period 11, actually making GAVE’s predicted brown line 11 much closer to the true line 11 than otherwise. In a similar way, line 9* affects the response of period 13.

The time difference of 448 days between the earlier and the actual weight that determines the weight velocity causes an even increased complexity of GAVE because brown lines 2, 5 and 7 in Fig. 2a also affect each other’s subsequent continuation, contributing to a much lower weight velocity during a much longer age period than perhaps expected, see brown lines 2–8, thus causing an even increased inaccuracy of GAVE predictions, see also the first incorrect prediction of GAVE during period 8 in the Discussion, sixth paragraph.

### Accuracy of the Response of GAVE for an Earlier vs. Later Weight Change

**Figure 3:**
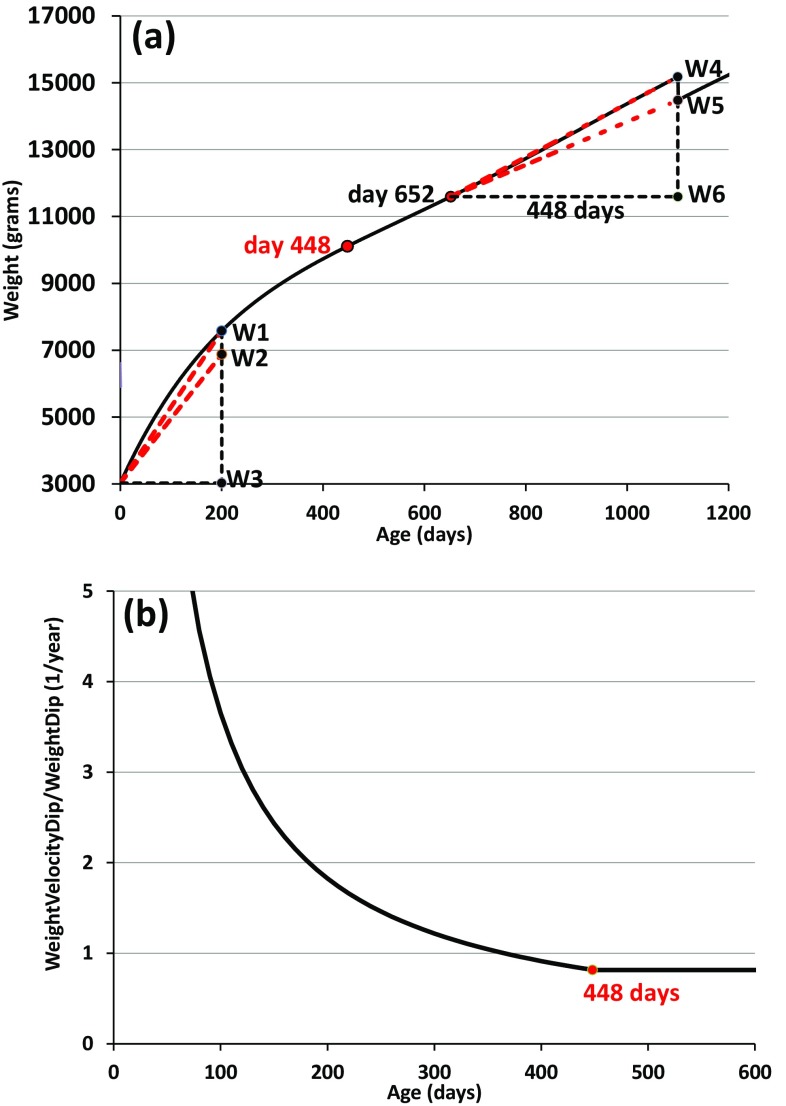
GAVE response to an earlier vs. later weight dip. (**a**) Weight velocity calculation by GAVE of one single abrupt dip of 700 g in the 0SD weight curve, assumed to occur individually at various ages. One example is shown before 448 days, at 200 days, weights indicated by *W*_1_ − *W*_3_ (*W*_3_ is birthweight) and one at *t* = 1100 days, indicated by *W*_4_ − *W*_6_. (**b**) The dip in GAVE’s weight velocity divided by the weight dip, according to Eq. (4).

To analyze the possible difference in inaccuracy of GAVE predictions as a function of age, we used the 0SD weight curve and added one single instantaneous weight dip of 700 g. We did that individually at various ages *t* until 1100 days of age. In Fig. 3a, we show the calculation of weight velocity in two situations, one in response to the weight dip occurring prior to 448 days, one occurring later. The weight velocity at weight *W*_1_, at age *t* of 200 days, is determined by the ratio (*W*_1_ − *W*_3_)/*t*, *W*_3_ being the birthweight. Weight velocity at *W*_2_ = *W*_1_ − 700 is similarly, (*W*_2_ − *W*_3_)/*t*. Because the weight velocity response by GAVE is proportional to the weight dip amplitude, we divide the difference between the two weight velocities, called *WeightVelocityDip*, by weight dip (*W*_1_ − *W*_2_) = 700 g as

4a $$ \frac{{\text{Weight\,velocity\,dip}}\;(t < 448)}{{\text{Weight\,dip}}\;(t < 448)} = \frac{{\frac{{(W_{1} - W_{3} )}}{t} - \frac{{(W_{2} - W_{3} )}}{t}}}{{(W_{1} - W_{2} )}} = \frac{1}{t}\;W_{3} \;{\text{is}}\;{\text{birthweight}}. $$

For ages *t *≥ 448 days, the weight at (*t* − 448), i.e., *W*_6_, functions as the origin of the Cartesian coordinate system, Fig. 3a, right upper part. Thus, using that *W*_4_ is the weight at *t* and W_5_ = W_4_ – 700 g, gives

4b $$ \frac{{\text{Weight\,velocity\,dip}}\;(t \ge 448)}{{\text{Weight\,dip}}\;(t \ge 448)} = \frac{{\frac{{(W_{4} - W_{6} )}}{448} - \frac{{(W_{5} - W_{6} )}}{448}}}{{(W_{4} - W_{5} )}} = \frac{1}{448}\quad t \ge 448\,{\text{days}}. $$

The results are presented in Fig. 3b where we expressed the age in years (448 days is 1.23 years). Thus, GAVE’s normalized weight velocity response to a weight dip is an inverse function of age until 448 days, followed by a constant value of 1/448 reciprocal days or, if age is expressed in years as was done in Fig. 3b, 1/1.23 = 0.815 reciprocal years. Equations (4) show that a weight dip occurring before 448 days of age has a significantly larger effect on the weight velocity predicted by GAVE than the same weight dip beyond that age, implying that the GAVE predictions have a larger inaccuracy at ages earlier than 448 days than at later ages.

## Discussion

Commercial computer program GAVE calculates erroneous weight velocities, for the clinical weight curve of Fig. 1b in all 14 age periods, and for the model example of Fig. 1c in 13 of the 14 periods, because GAVE is unable to account for abrupt changes in weight as well as abrupt but long-lasting changes in weight velocity. As a result, GAVE produces a puzzling mixture of multiple complex and interfering weight velocity patterns. We acknowledge that the clinical weight curve of Fig. 1b may be somewhat uncommon, however the weight configurations of the 14 periods may be more common, implying that GAVE, even when applied to regular weight curves, may still produce erroneous weight velocity patterns, e.g., demonstrated in Figs. 2a and 2b for the 0SD standard weight curve. The results in Fig. 2a and Table 1 also illustrate that GAVE-derived weight velocities can be significantly under- as well as overestimated. Particularly, changes in weight or weight velocity in the weight curve occurring about 448 days apart can produce very strong deviations from the true weight velocities, including inverse-echoes from weight changes that occurred 448 days earlier (e.g., Fig. 1c).

Unfortunately, comparison between weight velocities calculated by GAVE and other publications with weight velocity data is at best problematic for several reasons. First, weight curves are often called “growth” curves, despite that weight growth is obviously the derivative of the weight curve. Nevertheless, true weight velocities have been determined and published for many decades, e.g., already by Brandt,^2^ who assessed weight velocity from weekly and biweekly weight measurements. Second, age intervals between weight measurements differ significantly from the 448 days used by GAVE, they typically range from a few weeks to a few months. Interestingly, Ghaemmaghami *et al*.^9^ stated that “It is worth mentioning that – one year is too long for length and weight measurements during infancy, –”, a conclusion that is clearly confirmed by our results. A selection of weight velocity publications is presented hereunder.

Weight velocity standards from 1 to 3 months serial weight measurements for infants and children up to 20 years were published by Danner *et al*.^3^ Marinkovic *et al*. measured weights at birth, 1, 2, 3, 4, 6, 11, 14, 18, 24 and 36 months and used a fit to connect the weight data.^11^ Weight velocity was then determined by differentiation of the fitted curve, thus identical to what we did for the 0SD weight curve [Eq. (2)]. For very low birthweight hospitalized infants, growth velocity measurements are performed daily or weekly to adjust their fluid and nutrition.^4^,^7^,^6^ Ehrenkranz *et al*.^4^ used quadratic regression splines to fit the measured weights and to calculate weight velocities. Fenton *et al*.^6^ combined three very different formulas than our Eq. (1) to represent weight velocity with age intervals varying between 1 and 16 weeks, aimed to standardize growth velocity calculations of preterm infants. Interestingly, his publication on weight velocity during 21–41 days of life of very low birthweight babies (507–1440 g, for comparison, the − 2SD, Fig. 1b, birthweight is 2400 g) showed gigantic variation in the parameter used to represent weight velocity, by a factor of even ± 10 (!) when the time difference was 1 day and still by a factor of ± 1.3 when 7 days was used.^7^

Commercial available growth calculators seem to be rare. We identified two additional ones next to GAVE. First, the WINROP algorithm,^1^,^16^ standing for Weight Insulin-like Growth Factor Retinopathy Of Prematurity, which compares postnatal weekly measured weights in infants at risk for ROP with weights from infants that developed ROP. The WINROP algorithm has been simplified as an online monitoring system and can be accessed free of charge (https://winrop.com). Second, we have been informed that the Cerner Electronic Medical Record System has a commercial weight velocity calculator. However, we were unable to find any details because use of search term “Cerner AND weight velocity in infants” in CINAHL, EMBASE, PubMed and Web-of-Science gave no single hit.

The clinical case of Fig. 1b shows the remarkable feature that period 2 of Failure-to-Thrive started around 56 days, thus 433, almost 448 days earlier than the huge growth spurt (line 9) that started at day 489 (fourth hospitalization, see Appendix). This coincidence is the cause of the enormous inverse-echo indicated by lines 2* and 3* (Fig. 2a) that peaks at day 568 (= 120 + 448, day 120 is the last day of period 2). Furthermore, the strongly decreased weight velocities erroneously predicted by GAVE during periods 6 and 8 (Figs. 2a and 2b) are caused by the interfering influence of the various weight dips during these periods, all occurring within 448 days (Fig. 1c).

Two false predictions by GAVE prompted the boy’s third pediatrician, appointed 3.5 months after the separation period started and considering periods 8 and 10 only, to incorrectly confirm the diagnosis of malnutrition-based child abuse of the mother. These wrong predictions by GAVE are denoted first and second hereunder. First, GAVE-*predicted* weight velocities during period 8 at home, despite excessive caloric intake (see Appendix),^15^ were significantly *below* 0SD, 4.4–4.7 vs 6.4–4.8 kg/year (Fig. 2a); in reality it was the opposite, 5.8 vs. 3.3–2.7 kg/year (Fig. 2b). Second, GAVE-*predicted* weight velocities during the first 3.5 months of separation period 10, despite normal food intake,^15^ were significantly *above* 0SD, 5–5.9 vs. 4.8–3.5 kg/year (Fig. 2a); in reality they were virtually equal, 3 vs. 2.6 kg/year (Fig. 2b). Thus, these two erroneous predictions of GAVE falsely suggest that Rosenberg’s second criterion applied: “Separation of the child from the caregiver resolves the symptoms”, necessary to diagnose Pediatric Condition Falsification.^5^,^14^ In our opinion it is shocking that the third pediatrician trusted GAVE unconditionally without feeling any need to examine the actual weight curve.

Finally, the increase in weight of an infant is the highest in the first six month of life, see, e.g., Fig. 1a; it becomes gradually less strong thereafter, only to increase again with puberty. Decreased or absent weight gain during the first 6 months, for instance due to illness, will cause a larger drop in weight gain compared to the age period thereafter. A period of gastro-enteritis, i.e., vomiting and/or diarrhea (period 7), will also have a larger impact on the weight curve during the first year of life compared to later occurrences. The younger the infant is, the higher the percentage of body water. The water turnover by fluid intake and losses with urine and feces of an infant of six months is 800 mL/day or 150 mL/kg/day. The water turnover of an infant of 2 years is 1200 mL/day or 100 mL/kg/day. For comparison, the water turnover of an adult is around 30 mL/kg/day. A period of gastro-enteritis in a small infant will cause a much larger drop in weight compared to an infant of 2 years or an adult, because vomiting and diarrhea will add to the already existing high water loss. The time needed to regain the weight loss is also much longer in a small infant due to the high water turnover and lower capacity of the kidneys to reabsorb water.

In conclusion, the algorithm used by GAVE to compute weight gain velocity has major flaws. The source of invalid analyses by GAVE and also its exceedingly complex mixture of weight velocity patterns is that the weight gain is calculated over a too long period of time,^9^ 448 days or 1.23 years, a time period in which a child may easily have episodes of normal, rapid or even negative weight gain, see previous paragraph. In our case, it also contributed to the false accusation of child abuse and the separation of the infant from his parents with serious consequences for the boy and the family. Thus, until a new algorithm has been developed and validated, we recommend that the clinical use of this computer program for the purpose of weight velocity predictions should be discarded for children below 1.5 years of age, for cases where periods of sickness cause several weight dips within 448 days, and for cases that include a long-lasting change in weight velocity. An important lesson that can be learned from our analysis is to never use a computer program in medicine that has not been publicly described and lacks proof of validation.

## Appendix: Brief Summary of the Clinical Case

The clinical case is about a boy, the sixth child of normal parents, born at term with birthweight of 3.18 kg (P25 or − 0.6SD standard weight curve). Until 56 days of age, the boy moved towards the − 2SD weight curve (period 1, Fig. 1b). Hospitalization occurred during part of a period of negative weight gain, thus Failure-to-Thrive,^12^,^13^ which was caused by a cow milk allergy^15^ (period 2). Removing the cow milk proteins from the diet resulted in a significant increase in weight gain (period 3). In period 4, he was hospitalized again due to an erroneously assumed negative weight velocity but recommenced his positive weight gain albeit less strongly than previously. Period 5 included “stomach flu” and a negative weight gain. Period 6 showed catch-up weight growth and included the third hospitalization where cow milk allergy was confirmed. Period 7 showed a weight loss due to nine days of gastro-enteritis followed by a spectacular catch-up growth (period 8). Despite impressive above-average weight growth, the pediatrician erroneously believed that the boy grew insufficiently, and increased the food intake stepwise to three times normal, to be administered by gastric tube. Although not well tolerated and resulting both at home and during the fourth hospitalization in frequent and severe vomiting, a strong weight increase resulted (period 9). Inappropriately, two pediatricians accused the mother of purposely causing malnutrition of her boy, based on their erroneous assessment of insufficient weight velocity at home. The boy was placed in a foster home (period 10). The new appointed third pediatrician confirmed the diagnosis of Pediatric Condition Falsification solely based on an analysis using GAVE, explained in the Discussion, sixth paragraph, which led to an extension of the boy’s custody. During separation, the boy’s food intake normalized as did his weight velocity. Once the boy returned home to his parents again there was a significant increase in weight velocity (period 11). Period 12 included four weeks of airway infections and colds, as many others in that period, resulting in a weight loss, but resolution was followed again by positive weight velocities (periods 13 and 14). The boy has developed well albeit with susceptibility for nose colds.
